# The ethical significance of user-control in AI-driven speech-BCIs: a narrative review

**DOI:** 10.3389/fnhum.2024.1420334

**Published:** 2024-06-27

**Authors:** O. C. van Stuijvenberg, D. P. S. Samlal, M. J. Vansteensel, M. L. D. Broekman, K. R. Jongsma

**Affiliations:** ^1^Department of Bioethics and Health Humanities, Julius Center for Health Sciences and Primary Care, University Medical Center Utrecht, Utrecht University, Utrecht, Netherlands; ^2^Department of Philosophy, Utrecht University, Utrecht, Netherlands; ^3^Department of Anatomy, University Medical Center, Utrecht University, Utrecht, Netherlands; ^4^University Medical Center Utrecht Brain Center, Department of Neurology and Neurosurgery, University Medical Center Utrecht, Utrecht, Netherlands; ^5^Department of Neurosurgery, Haaglanden Medical Center, The Hague, Netherlands; ^6^Department of Neurosurgery, Leiden University Medical Center, Leiden, Netherlands

**Keywords:** ethics, brain-computer-interface, artificial intelligence, locked-in-syndrome, speech, neuroethics, paralysis

## Abstract

AI-driven brain-computed interfaces aimed at restoring speech for individuals living with locked-in-syndrome are paired with ethical implications for user’s autonomy, privacy and responsibility. Embedding options for sufficient levels of user-control in speech-BCI design has been proposed to mitigate these ethical challenges. However, how user-control in speech-BCIs is conceptualized and how it relates to these ethical challenges is underdetermined. In this narrative literature review, we aim to clarify and explicate the notion of user-control in speech-BCIs, to better understand in what way user-control could operationalize user’s autonomy, privacy and responsibility and explore how such suggestions for increasing user-control can be translated to recommendations for the design or use of speech-BCIs. First, we identified types of user control, including executory control that can protect voluntariness of speech, and guidance control that can contribute to semantic accuracy. Second, we identified potential causes for a loss of user-control, including contributions of predictive language models, a lack of ability for neural control, or signal interference and external control. Such a loss of user control may have implications for semantic accuracy and mental privacy. Third we explored ways to design for user-control. While embedding initiation signals for users may increase executory control, they may conflict with other aims such as speed and continuity of speech. Design mechanisms for guidance control remain largely conceptual, similar trade-offs in design may be expected. We argue that preceding these trade-offs, the overarching aim of speech-BCIs needs to be defined, requiring input from current and potential users. Additionally, conceptual clarification of user-control and other (ethical) concepts in this debate has practical relevance for BCI researchers. For instance, different concepts of inner speech may have distinct ethical implications. Increased clarity of such concepts can improve anticipation of ethical implications of speech-BCIs and may help to steer design decisions.

## 1 Introduction

Locked-in syndrome (LIS) is characterized by a loss of voluntary muscle control, resulting in quadriplegia and anarthria, yet patients retain normal cognitive function (American Congress of Rehabilitation Medicine, 1995; Smith and Delargy, 2005). LIS can be caused by events such as a brainstem stroke and motor neuron diseases like amyotrophic lateral sclerosis (ALS). While significant debilitation and progressive decline in individuals with ALS have been reported to negatively impact standardized health-related quality of life (QoL) (Neudert et al., 2004; Peseschkian et al., 2021), individual subjective QoL was found to be independent of general (declining) physical function, to remain stable, and to be relatively high (Robbins et al., 2001; Neudert et al., 2004; Kuzma-Kozakiewicz et al., 2019). In a longitudinal study including individuals with LIS due to stroke, relatively satisfactory and stable self-reported QoL was similarly observed (Rousseau et al., 2015). Factors that were reported to play a role in individual self-reported QoL in LIS patients included factors like family, social life and relations, health, profession, spirituality, religion and psychological factors (Robbins et al., 2001; Neudert et al., 2004). In line with these factors, unlike general physical functions, the ability to (verbally) communicate with family members and caregivers was argued to be critical in maintaining QoL in individuals with LIS (Liberati et al., 2015; Felgoise et al., 2016).

At present, communication methods for individuals with LIS encompass various augmentative and alternative communication (AAC) approaches. These include no-tech strategies such as eye movements or blinking to respond to yes/no questions, low-tech solutions involving communication partners (e.g., using letter boards, static devices) and high-tech solutions (e.g., eye-tracking devices) (Vansteensel et al., 2023). While these methods provide valuable options for communication, they also have limitations, including the speed of communication, flexibility/variability of communication, and the requirement of functioning eye-movement (Luo et al., 2022). Vansteensel et al. (2023) for instance discuss how eye-tracking devices are not usable for a significant portion of people with late stage ALS due to difficulty in maintaining stable head position (Spataro et al., 2014), pupil dilation because of Baclofen use (Chen and O'Leary, 2018), progressive oculomotor impairment and eye-gazing fatigue (Spataro et al., 2014; Nakayama et al., 2015).

Implantable brain-computer interfaces (BCIs) have been proposed as a new potential high-tech AAC approach for people with LIS, or others living with (paralysis and) anarthria (Wolpaw et al., 2002; Vansteensel and Jarosiewicz, 2020). A BCI can be defined as “a system that records [central nervous system (CNS)] activity and translates it into artificial output that replaces, restores, enhances, supplements, or improves natural CNS outputs; it thereby modifies the interactions of the CNS with the rest of the body or with the external world” (Wolpaw et al., 2020, p. 16). A BCI aimed at restoring speech (i.e., a speech-BCI) thus aims to decode neural signals correlated to speech intentions from the motor cortex and translates these signals to written text or synthetic speech on a computer.^1^ The interpretation (i.e., decoding and translation) of these complex neural signals is performed using predictive algorithms, including language models (Zhang et al., 2020). Recent speech-BCIs developed by Metzger et al. (2023) and Willett et al. (2023) have shown significant advances both in speed and accuracy of BCI-mediated speech. These studies showed decoding of 78 words per minute with a 25% word error rate using a 1,024 word vocabulary (Metzger et al., 2023), and of 62 words per minute with a 23.8% word error rate using a 125,000 word vocabulary (Willett et al., 2023).

The aim of the development of such artificial intelligence (AI-)driven speech-BCIs is to improve quality of life (QoL) of users by further improving their autonomy. This can, for instance, be achieved through optimizing the user’s functional independence in communication (e.g., by being less dependent on the interlocutor for initiating and steering conversation in settings of daily living) and in terms of self-determination (e.g., by facilitating communication and articulation of autonomous decisions in the context of medical treatments, clinical trial participation, end-of-life, and personal situations) (Glannon, 2022).

Yet, while speech-BCIs offer important opportunities for, for example, LIS patients, BCIs are also paired with ethical implications. These include implications for user’s autonomy, privacy, and responsibility (Burwell et al., 2017). Embedding options for sufficient levels of user-control in speech-BCI design has been proposed to mitigate these ethical implications (Bublitz et al., 2019; Kreitmair, 2019; Maslen and Rainey, 2021; Sankaran et al., 2023). For the purpose of this review, we define user-control in speech-BCIs as users being able to implement if, what and how BCI-mediated synthetic speech is produced, according to their intentions. In this way, user-control may protect the voluntariness of synthetic speech and prevent the production of synthetic speech that does not match the user’s intentions. However, operationalizing user’s autonomy, privacy and responsibility through increased user-control may not be straightforward. For example, controlling what, if or how synthetic speech is produced each may have different implications for users’ autonomy and responsibility. Also, if predictive language models are used to decode neural signals, they also provide a contribution to BCI-mediated speech, which may be considered as exercising some type of control.

Therefore, in this narrative literature review, we aim to clarify and explicate the notion of user-control in speech-BCIs, to better understand in what way user-control could operationalize user’s autonomy and responsibility and explore how such suggestions for increasing user-control can be translated to recommendations for the design or use of speech-BCIs. We first discuss how user-control can be conceptualised by defining two types of control. Then, we explore multiple ways in which user-control may be compromised and discuss related ethical implications. Next, we discuss ways in which user-control may be embedded in speech-BCI design. Last, we make suggestions for future research.

## 2 Types of user-control

Considering user-control in speech-BCIs, two types can be distinguished: executory control and guidance (or process) control.

### 2.1 Executory control

Executory control in speech-BCIs firstly concerns itself with the control over *if* something is spoken (Maslen and Rainey, 2021). In practice, such control could constitute control over whether a decoder is engaged (Sankaran et al., 2023), or as a trigger to ‘turn on’ the device (Rainey et al., 2019). As such, executory control is essential for the protection of voluntariness or externalized speech. In their seminal article on control and ownership in speech-BCIs, Maslen and Rainey (2021) consider goal selection (i.e., selecting a particular action for initiation) to be a second component of executory control. In this way, executory control also covers the choice for externalization of *what* speech, not ‘any’ or random speech. Steinert et al. (2019) describe that goal-selection in executory control can be considered as a type of ‘auto-pilot’, as users do not have any further control on how this goal is reached or to adjust the process (i.e., lacking guidance control) (Steinert et al., 2019).

Important to note here, however, is that a precondition for users to be able to exercise any control over speech-BCIs is that the device is sufficiently technically accurate (Maslen and Rainey, 2021), irrespective of the specific speech-BCI decoding approach. Maslen and Rainey (2021) define technical accuracy as there being “a close correspondence between neural activity, the recording made by the neuroprosthetic device, the digital representation of the recording, and the synthetic speech output, in a pairwise fashion.” (p. 430). The technical accuracy of speech-BCIs could be impeded by various factors. The device could fail to detect or misinterpret neural signals, and artifacts originating from physiological or environmental processes can obscure or contaminate signals underlying output commands (Bublitz et al., 2019; Wolpaw et al., 2020). For example, producing speech and perceiving speech have overlapping or coexisting neural properties. That is, the sensorimotor cortex, which is targeted by the speech-BCI for decoding motor-intentions for speech, is also engaged during perception (i.e., hearing) of speech. In practice this means that the BCI may detect neural signals that correspond to what the user hears, rather than to speech they intend to produce. This makes that speech perception could interfere with reliable speech-BCI control (Rainey et al., 2019; Sankaran et al., 2023). To add to the complexity, BCI systems do not provide users with direct feedback on such errors, making that in practice it may be hard to distinguish between sources for inaccuracies in BCI-mediated speech (Bublitz et al., 2019).

### 2.2 Guidance control

In speech-BCIs that aim to establish continuous speech, executory control, addressing *if* and *what* is said, could be considered somewhat ‘coarse grained’ (Rainey et al., 2019). To facilitate complex continuous conversational speech, a particularly high degree of control will be required (Maslen and Rainey, 2021). Guidance control, or process control, is such a high level of control as this could contribute to *how* speech externalized, allowing users to continuously alter and influence the speech they produce (Maslen and Rainey, 2021). Guidance control would thus go beyond selecting goals and initiating processes as done by executory control but would rather govern the implementation of speech (Rainey et al., 2019; Wolpaw et al., 2020; Maslen and Rainey, 2021).

Guidance control in BCIs is argued to be important for applications where goals are not fully defined or have multiple possibilities, in scenarios where unexpected complications can occur, or for users seeking maximum control (Wolpaw et al., 2020). Speech-BCIs that aim to establish continuous speech, similar to healthy speech, match these criteria because of the indeterminate nature of speech. In continuous and spontaneous speech, namely, a speaker does not predefine and command a clear goal. Rather, intentions for speech continuously and dynamically unfold as we speak, the content of continuous speech becoming apparent during the act of speaking (i.e., thinking as we speak) (Maslen and Rainey, 2021). Maslen and Rainey (2021) discuss these “hazy intentions” as also existing in the covert speech that could be used to control speech-BCIs (p. 432). As predictive language models in these devices will attempt to ‘fill in the gaps’ caused by this indeterminacy of speech intentions, it may become more difficult for users (as well as for developers) to recognize the *semantic accuracy* of BCI-mediated externalized speech (Maslen and Rainey, 2021; Rainey, 2022). Semantic accuracy can be defined as “how well it discloses the content or meaning the user intends to communicate” (Rainey et al., 2019, p. 661). Considering the expressive nature of speech, semantic accuracy is likely to be important for user satisfaction with continuous speech-BCIs (Rainey et al., 2019).

## 3 Loss of user-control

To explore the ethical significance of these types of user-control in speech-BCIs, we here explore the ethical implications of a loss of user-control. In this paragraph we discuss three potential causes for a loss of user-control and address related ethical implications. First, we address shared control with predictive language models, next we discuss users’ lack of ability for neural control, and third we discuss loss of user-control caused by signal interference.

### 3.1 Contribution of predictive algorithms leading to shared control

As discussed in relation to guidance control, a first cause for loss of user-control can be found in speech BCI-technology itself: in the predictive algorithms embedded in the device. AI in BCIs is argued to increase accuracy, reliability, adaptability and speed of information processing (Karikari and Koshechkin, 2023). Reconstructive and predictive algorithms that partly use statistical language models can be embedded in speech-BCIs to enable and improve the quality and efficiency of the interpretation of, sometimes imperfect, neural signals (Maslen and Rainey, 2021). In performing these tasks, predictive algorithms provide a contribution to, or ‘a shaping of’, the output. This contribution is beyond the user’s control, leading to a level of *shared control* between users and these systems (Maslen and Rainey, 2021; Glannon, 2022). This type of shared control thus necessarily introduces a limitation to user-control. The extent of this limitation depends on the distribution of shared control between the entities (i.e., the system and the user). A system with a high level of reconstruction and prediction (i.e., contribution) necessarily creates a larger limitation to user-control. Important to note here is that contributions of predictive language models also exist in other existing AACs. Some of these discussions on shared control therefore may also apply to these technologies.

Limitations to user-control due to shared control with predictive algorithms may lead to differences between user’s speech intention and the externalized BCI mediated speech, which can have implications for user’s self-expression and self-representation [or authenticity (Rainey et al., 2019)]. Self-expression may be harmed when users are not able to express themselves through the language they intended. Self-representation of users may be harmed if mistakes are made in algorithmic predictions, and this erroneous speech is taken as that of the user (Maslen and Rainey, 2021). Maslen and Rainey (2021) argue that this would challenge user’s “first-person authority” (p. 2305). They also argue that, given the necessary limitation to user-control in case of shared control “the most pressing ethical question will be whether the user sufficiently endorses the output” (p. 430), which they define as ‘ownership’.

Theoretically, users themselves could also endorse slightly erroneous speech and accept it as their own, especially if their speech intentions were particularly “hazy” to begin with. It is not likely that users this way would accept BCI-mediated speech that is very far from their intended speech, but they may accept some of the predictions made by the predictive language models to ‘fill in the gaps’ that are close enough for them to accept but would not be an exact match to their intentions if these had been more determined (e.g., “Yes, I guess that’s what I meant!”). In this way predictive algorithms in speech-BCIs could influence users’ thinking, or how they perceive themselves (e.g., “I did say that, and now I think of it, I actually do stand by it!”). Klein et al. (2022) similarly discuss how language, words, and phrases one uses for expression reflect and shape one’s identity. In their qualitative study with potential users of speech-BCIs, they found that personalization of language models therefore was considered to have the potential to affect user’s identity. They report that respondents wanted a language model that could allow them to express their personality and “communicate in familiar and valued ways to *them*” (Klein et al., 2022, p. 6). This was considered of particular importance in the social context such as communication with loved ones and intimate partners and for maintaining various other social relationships, yet respondents also expressed privacy concerns in case personalized language models “reveal too much” (Klein et al., 2022, p. 7). To note is that these concerns on predictive algorithms steering users’ thinking are not limited to speech-BCI technology and may also exist for users of other types of predictive algorithms, including link recommendations in social media (Santos et al., 2021), or language-model-powered writing assistants (Jakesch et al., 2023).

Shared control and the role of neural signal processing in speech-BCIs also has implications for the user’s responsibility for output (Rainey, 2022). Regarding legal responsibility, Bublitz et al. (2019) discuss the complexity that arises when a user and BCI-system become “physically inseparable and functionally interwoven” (p. 1). They argue that in case of joint output (i.e., through shared control), it becomes challenging to distinguish the separate contributions of each entity. Still, the contribution of predictive algorithms in shared control of a speech-BCI does make that there is at least a possibility that speech is produced for which the user is not responsible. However, even though this contribution of predictive language models may absolve users of responsibility of BCI-mediated speech, the BCIs themselves are not entities that are suitable for attribution of blame and liability either (Bublitz et al., 2019). Moreover, also in the context of informed consent procedures, contributions to the ‘shaping of’ BCI-mediated speech by predictive language models may lead to slight departures from user intentions. This contribution may for instance raise questions on the validity of informed consent provided. This example is particularly intriguing as well as ethically problematic, as speech-BCIs are also described as a tool for assessing or restoring the capacity to give informed consent in people with LIS (Brukamp, 2013; Klein et al., 2018). Glannon (2022) describes how in decisions about continuing or discontinuing life sustaining treatment, the ethical stakes of reliable BCI-mediated communication are particularly high.

While shared control in speech-BCIs may have implication for users’ self-expression, self-representation, self-perception, and responsibility, it also has significant benefits. Klein et al. (2022) [referring to Newell et al., 1998] write that the goal of adding natural language processing models to communication technology is “to (1) reduce the physical input necessary to produce an utterance; (2) reduce the cognitive load on the user; (3) increase the speed of communication; and (4) reduce the delays between the user formulating what they want to say and the device articulating the appropriate words” (p. 1). Glannon (2022) argues that shared control in fact realizes the rehabilitative and restorative potential of speech-BCIs, and that the current state of these technologies “does not indicate that implants and algorithms control the minds and behavior of people in whom they are implanted or to which they are connected.” (p. 17). Additionally, Sankaran et al. (2023) point out that we should consider the use of predictive language models in speech-BCIs in the context of (current) speech-decoding capabilities. They argue that at present unaided neural inference of speech remains imperfect, limiting speech-BCI users in authoring and transmitting messages according to their intentions. They argue that language models would therefore present (drastic) gains in accuracy of output, rather than a loss (Sankaran et al., 2023).

### 3.2 Lack of ability for neural control

A second cause for a loss of user-control in speech-BCIs can be found in the users themselves, in a potential lack of ability for neural control. If users lack the ability to control their neural activity related to their motor speech intentions in a precise and intentional way, this may affect the distribution of (shared) control, requiring embedded algorithms to make more predictions and reconstructions (Maslen and Rainey, 2021). However, “the quality and consistency of the data can affect the performance of the AI algorithm, as inaccurate or inconsistent data can lead to incorrect predictions and unreliable results” (Karikari and Koshechkin, 2023, p. 1356).

A lack of ability for neural control in speech-BCIs may have physical causes, as it is uncertain if and how specific medical conditions might limit some users’ ability to exercise such control. Carmichael and Carmichael (2014) described how “BCI illiteracy” [which is a contested term signifying the users’ inability to use a BCI device despite multiple test sessions (Thompson, 2019)] could be associated with symptoms of an underlying condition, in particular reduced information processing speed. Vansteensel et al. (2023), similarly stated that users “will need to be prepared for the possibility that BCI performance may be affected by progressive disease, changes in the electrode-tissue interface, or neural plasticity influencing BCI control signals” (pp. 1330–1331). Moreover, it has also been argued that complete LIS (CLIS) may impede user-control in speech BCIs (Birbaumer et al., 2014; Poppe and Elger, 2024). Birbaumer et al. (2014) suggested that thought extinction and alternative sleeping patterns in CLIS could lead to fading vigilance during BCI-based communication training, hampering the learned ability for control. However, a more recent study actually showed decent, albeit intermittent, BCI control by an individual with CLIS using auditory feedback training, suggesting that BCI control is possible even in a complete locked-in state (Chaudhary et al., 2022).

A lack of ability for neural control of speech-BCIs due to physical causes may limit access of specific patient groups to this technology. Becker et al. (2022) argue that the issue of “BCI illiteracy” may be improved by better taking into account in the BCI development process the variability between prospective users, including their individual expertise, the variability in brain structure and variability in cognitive strategy.

Ideally a BCI system should be sufficiently robust to identify poor user ability to modulate neural signals related to speech intentions. It is to some extent possible to check whether users can modulate neural signals, e.g., by asking them to speak certain words and seeing if the decoder identifies the correct phonemes or words. Yet, in case problems occur, it may initially be difficult to determine whether these errors originate from (incorrect) neural signals generated by the user, or by the decoder. However, if a decoder initially did function well, but errors start to occur at a later stage, this may indicate that something has changed in the underlying neural signals.

### 3.3 Signal interference: external control and suboptimal decoders

Thirdly, a loss of user-control in speech-BCIs can also occur because of signal interference in the BCI-system, which may have different causes.

Signal interference may firstly be caused by external control [or external interference (Glannon, 2022)] over speech-BCIs, which can exist in different forms. A first form of external control over speech-BCIs that could cause signal interference could lead to the externalization of speech that is fully independent of the user’s neural signals, does not originate from any (motor) speech intention, nor correlates to inner speech of the user. Such ‘complete’ external control could occur when an external entity has full control over the speech BCI and overrides the BCI’s signals to the output device (e.g., in case of hacking) (Ienca and Haselager, 2016, Karikari and Koshechkin, 2023). Glannon (2022) argues that wireless design of BCIs may increase risks for such remote (i.e., external) control. A second form of external control that could cause signal interference could lead to the involuntary externalization of speech. Such involuntary externalization could, for instance, similarly occur in a case of hacking of the system, or in case of remote programming by physicians (Glannon, 2022). Rainey et al. (2019) discuss that such involuntary externalization could concern speech that was not “1) intended to be externalized at all (e.g., private thought), 2) ready to be externalized (e.g., reflection, ‘practice’ or ‘preparatory’ speech), 3) externalized as was intended (e.g., malapropism), or 4) intended at all (e.g., mind-wandering)” (p. 2306).

In addition to external control, signal interference may also be caused by suboptimal decoders in the device. Suboptimal decoding may similarly result in involuntary externalization of speech when systems are triggered prior to the user’s conscious decision to act (Glannon, 2016; Rainey et al., 2019, 2020a; Maslen and Rainey, 2021). This could for instance occur when neural signals other than those related to motor intentions for speech are mistaken for speech attempts and are decoded and externalized by the device. Though this situation is different from external control by hacking or external programming in the sense that there is no external entity actively attempting to control the device, it does signify a situation whether the locus of control over externalization of speech is not with the user and where a system is undertaking delegated action without user’s permission (Bocquelet et al., 2016; Glannon, 2016; Rainey et al., 2020a).

The ethical implications of these types of signal interference, and particularly the possibility for involuntary externalization of speech, are a prominent topic in discussion on ethics of (AI-driven) speech-BCIs. Similar to shared control with predictive algorithms, the potential possibility of signal interference in speech-BCI raises questions on users’ responsibility for BCI-mediated speech. Naturally, if output generated through ‘complete’ external control is taken as that of the user, this can have serious consequences depending on the context (e.g., in medical decision-making). However, signal interference leading to involuntary externalized speech also raises questions on responsibilities for BCI-mediated speech, and whether users should be judged based information that is shared voluntarily or on what can be inferred from (involuntarily) decoded inner speech (Rainey et al., 2020a). Scenarios involving such questions on responsibilities could arise when users involuntarily externalize speech that has legal, social, or medical implications. For example, confessing to a crime, being rude or insulting to loved ones or care partners, expressing significant medical or (end-of) life decisions, or providing informed consent. In the context of informed consent, for instance, involuntary externalization of speech could potentially lead to questions on the user’s true wishes. Especially if the device externalizes a part of the user’s inner monologue that differs from their own controlled externalized speech, this raises questions on what would constitute respecting the user’s autonomy, introducing a risk for user’s self-determination. Addressing these uncertainties, it has been suggested that users’ responsibility for BCI-mediated speech correlates to the level of control the user had and the foreseeability of the given output (Rainey et al., 2019; Steinert et al., 2019; Maslen and Rainey, 2021). Additionally, it has been argued that such issues on responsibility should guide and the impact the development and design of these devices (e.g., by ensuring voluntariness of externalized speech) (Bublitz et al., 2019; Rainey et al., 2020b; Maslen and Rainey, 2021).

One of the most prominent and widespread ethical debates regarding speech-BCIs concerns the implications of involuntary externalization of BCI-mediated speech for users’ mental privacy and cognitive liberty. Rainey et al. (2020a) argue that “One of the main conceptual, technological, and ethical difficulties here is to distinguish the covert speech that should be externalized from that which should not” (p. 2305). Mental privacy has been defined as “explicitly protect[ing] individuals against the unconsented intrusion by third parties into their mental information (be it inferred from their neural data or from proxy data indicative of neurological, cognitive, and/or affective information) as well as against the unauthorized collection of those data” (Ienca, 2021, p. 5). To this definition we would add that mental privacy should also protect individuals against unconsented intrusion (i.e., involuntary externalization) caused by suboptimal decoders, as described above. Harms to mental privacy could introduce a major shift from how we think about one’s mind: as only accessible to oneself (Rainey et al., 2020a). According to Rainey et al. (2020a) studies using identification algorithms on fMRI data seem to show that mental content can be extracted directly from brain measurements, which means that the conviction that we have exclusive access to our thoughts is misplaced. Though current BCIs do not allow for this, in theory, this could mean that an external entity, or other person, may access, among others, ideas, emotional states or memories of a BCI user, without their express permission (Rainey et al., 2020a). In unaided and unimpaired communication, individuals can choose to provide such access to conversation partners, both verbally and non-verbally. However, the main difference here is that in such communication individuals are in control of providing such access. Yet, such control is lost in case of signal interference in BCI-mediated speech, potentially harming mental privacy. Such a breach of mental privacy could also impact users’ cognitive liberty, which can be defined as “a person’ autonomous, unhindered control or mastery over their mind” (Ligthart et al., 2023, pp. 467–468). A breach of mental privacy could threaten cognitive liberty as users may not feel free to think, reflect and deliberate without threat of consequences, and may harm user’s agency and self-representation (Rainey et al., 2020a). An important example could be found in (medical) decision-making, which requires users to reflect on options, potential futures, fears, desires and needs, which can be highly personal. If such a personal reflective practice becomes accessible to others, users may not feel free to consider and choose certain options. Also, in the context of social and intimate relations, limitations to mental privacy and cognitive liberty could be harmful. Kreitmair (2019) argues that risks of involuntary externalization “would likely render the technology less attractive to users, who might perceive it as a violation of the integrity of their private inner worlds, which healthy individuals take for granted” (p. 671).

As a response to implications of neurotechnologies for mental privacy and cognitive liberty, propositions have been made for the development of neurorights (Ienca and Andorno, 2017, Yuste et al., 2017, 2021, Ienca, 2021). Neurorights can be defined as “the ethical, legal, social, or natural principles of freedom or entitlement related to a person’s cerebral and mental domain; that is, the fundamental normative rules for the protection and preservation of the human brain and mind” (Ienca, 2021, p. 6). The development of such new legislation has been argued to be necessary as human rights would not be normatively sufficient to respond to the emerging issues raised by neurotechnology (Ienca and Andorno, 2017; Ienca, 2021). Still, though there appears to be consensus that neurotechnology users should receive sufficient legal protections, there remains scholarly disagreement on whether a new legislative framework (i.e., neurorights) is necessary to ensure such protection, or that these protections can already be explained as a new interpretation of existing human rights (Ligthart et al., 2023).

## 4 Design for user-control

We have so far established types of user-control, ways in which user-control can be lost and ethical implications that may follow. Executory control in users over BCI-mediated speech can protect voluntariness of speech, mitigating risks for self-representation, self-determination, responsibility, mental privacy and cognitive liberty. In addition, by influencing how BCI mediated speech is externalized, guidance control in users can improve self-representation, self-determination and responsibility as well. To allow for these types of user-control, several suggestions have been made to embed control-mechanisms in speech-BCI design.

### 4.1 Design for executory control

Assuming that the precondition of technical accuracy has been met, several suggestions are made to improve executory user-control in speech-BCIs by embedding certain mechanisms in their design. A first general claim is that it is important to achieve sufficient levels of both sensitivity and specificity in speech BCIs (Sankaran et al., 2023). Sensitivity is required to detect neural signals for attempted speech in absence of motor-abilities for speech, and specificity is needed to only decode such neural signals when intended (Sankaran et al., 2023). More specific mechanisms for executory control are mechanisms that allow users to give an initiation (or ‘go’) signal. These signals could be used to distinguish speech intended for externalization from other types of inner speech and allow for the retraction of incorrect speech (Maslen and Rainey, 2021). Authors also discuss what type of physical signal users could use to signify such a go-signal (Kreitmair, 2019; Sankaran et al., 2023). Sankaran et al. (2023) propose that to promote agency in users a speech-BCI should include “a separate speech-detection module that functions prior to decoding speech content in order to identify the temporal onset and offset boundaries of intended speech” (p. 2). That is, there should be a control signal to tell the BCI when to start, and when to stop decoding, that is separate from the detection of the intended speech itself. Sankaran et al. (2023) argue that in principle any attempted command that reliably generates a salient signal could be used to comprise such a start or stop signal yet argue that non-speech motor attempts would be preferable as these may be more easily distinguished from neural signals corresponding to attempted speech. Attempted hand movements can be used as switch signals for BCI users (Vansteensel et al., 2016; Oxley et al., 2021), and it was recently found that they can also be reliably decoded by a speech-BCI while remaining highly distinguishable from attempted speech (Metzger et al., 2022, 2023; Sankaran et al., 2023). Additionally, Kreitmair (2019) explores the possibility of users thinking of a particularly salient mental image or sentence, or another detectable dimension of thought, to trigger externalization. Examples of such triggers (or go-signals) are, for instance, Apple AI and Amazon AI that use the words “Siri” and “Alexa” to allow for a particular functionality “to spring into action” (Kreitmair, 2019, p. 671). Kreitmair (2019) does note that such neural control mechanisms likely require considerable training for both the user and algorithm, though if successful, could lead to an organic way of user-control. Alternatively, physical signals such as eye gaze could potentially also be used as go-signals (Sankaran et al., 2023). However, whether such a control signal is suitable does depend on whether a user is (still) able to generate the selected physical signal in a reliable fashion, which may vary between users.

In addition to the type of neural of physical command that can signify a go-signal, the moment on which such a signal can be given could vary. To start, a go-signal could follow a ‘first-listen’ or ‘first-read’ approach, so that users can evaluate drafted speech and consequently decide whether to externalize it (Rainey et al., 2019; Sankaran et al., 2023). Though such approaches would ensure executory control, they would also significantly limit the speed and continuity of BCI-mediated speech (Rainey et al., 2019). So, while different neural and physical mechanisms for executory control signals may be possible, these should be adapted to specific users as their capabilities and preferences for control signals may vary.

### 4.2 Design for guidance control

While conceptually allowing for guidance control in speech-BCIs may seem straightforward, it is not clear what could constitute a mechanism for guidance control in speech-BCI design. Though the user’s initial motor intentions for speech may be considered a means to exercise some guidance control (after all these may include intentions on *how* this speech is to be externalized), interpretation of the (often imperfect) corresponding neural signals by predictive language models may fail to capture these (nuanced) intentions. Means for exercising guidance control after the stage of interpretation by predictive language models may therefore be necessary to maintain semantic accuracy.

At first glance, a mechanism for guidance control in speech-BCIs would at least require a type of feedback and review mechanism through which users can evaluate the interpretation by predictive language models. Yet, if such a review process would be ‘static’ (i.e., to review proposed speech at a specific time before externalization), this would rather constitute a mechanism for executory control (i.e., the first-listen approach). Alternatively, a mechanism for a type of ‘veto-control’ could be used to allow users to stop speech in its track when users notice (semantic) mistakes (Maslen and Rainey, 2021). Yet, it is questionable whether such a (post-hoc) veto signal would signify actual guidance control (Maslen and Rainey, 2021), or whether it may be considered as a separate type of control (Steinert et al., 2019), or simply as a negative form of executory control. Moreover, discussing the possibility of such retractions and corrections during BCI-mediated speech, Sankaran et al. (2023) also warn that in speech BCIs that decode and externalize speech with low-latency, externalized speech may coincide with new speech attempts (e.g., the following sentence), making such error-corrections more difficult. Moreover, for fine-grained guidance control, such a review process should rather be ‘continuous’: a continuous, or incremental, review stage(s) parallel to the production of speech to allow users to steer, moderate and curate externalized speech. This would require users to receive continuous feedback during speech. One type of such feedback could be immediate auditory feedback (i.e., hearing what you say), which plays an important role in the speech production process (Guenther and Hickok, 2015; Luo et al., 2022). In unimpaired speech production also other types of sensorimotor feedback exist during self-perception (Kröger et al., 2019). Though it should be further researched which types of feedback could exist in BCI-mediated speech (e.g. in individuals with LIS), such continuous feedback and review would probably require even higher-speed decoding of neural signals.

In line with this ‘need for speed’ Maslen and Rainey (2021) argue that “the more automated the [correction and retraction] process, the more valuable this will be” (p. 438). Paradoxically, a requirement for high-speed decoding and high levels of automation would likely require a more significant contribution of predictive algorithms in continuous speech-BCIs, again risking that continuous BCI-mediated speech is steered away from users intended speech.

## 5 Future research

### 5.1 Trade-offs in design: preceding considerations

In order to address concerns about self-representation, self-determination, responsibility, mental privacy and cognitive liberty in speech-BCI design, it is important that these should be considered in context of the consequential trade-offs with other aims of speech-BCI, such as the speed and continuity of BCI-mediated speech. We would however argue that while concrete trade-offs between such aims are necessary to guide design, an overarching consideration that should precede these trade-offs is the definition of the overall aim of speech-BCIs themselves, and whether these technologies should be considered as expressive, or instrumental. That is, is the aim of speech-BCIs to allow users to express themselves through synthetic speech to the same extent as able-bodied individuals, or, are speech-BCIs considered tools for more functional types of communication such as decision-making? This consideration is important here as, for instance, implementing measures for guidance control in speech-BCIs that aim to produce spontaneous and continuous conversational speech can prove to be particularly challenging, as any review stage would reduce speed and continuity. In speech-BCIs less aimed at achieving continuous speech, a ‘first-listen-approach’ may be a perfectly acceptable way of improving semantic accuracy of speech. Additionally, whether a device is considered expressive or instrumental may influence the question on “how close is close enough?” in externalized speech (i.e., what is the relative importance of a high level of semantic accuracy?). Moses et al. (2021) for instance report that “speech-decoding approaches generally become useable at word error rates below 30%” (p. 226). Though an error rate of this size may suffice for usability, it does not meet criteria for semantic accuracy. Yet, Maslen and Rainey (2021) argued that semantic accuracy in BCI-mediated speech was of particular importance because “speech is inherently expressive and indirectly (and often secondarily instrumental)” (p. 433). Rainey (2023) recently concluded that “Synthetic speech systems may be best thought of as tools implementing novel communicative practices modeled on the familiar, not as technically mediated continuations of the familiar” (p. 8).

Considering the overarching, as well as more concrete, trade-off(s) in speech-BCI design, an increasing number of scholars have argued for the inclusion of perspectives of end-users, for instance via user-centered-design approaches (Huggins et al., 2011; Kübler et al., 2014; Liberati et al., 2015; Johansson et al., 2017; Branco et al., 2023; Sankaran et al., 2023). The inclusion of user perspectives can contribute to the development of a BCI that is fully accepted by users and meets user needs (Huggins et al., 2011; Branco et al., 2023). For people with LIS who have lost a considerable degree of autonomy and physical liberty, it could be imagined that cognitive liberty and mental privacy become increasingly important to their quality of life, justifying the need for, at least, high levels of executory control. However, determining the actual importance of executory and guidance control relies on the wishes and desires of (future) potential users, which can only be ascertained through empirical studies of preferences. Schicktanz et al. (2015) for instance describes how patients as opposed to experts hardly problematize questions on stability, consistency and authenticity of personal identity and Branco et al. (2023) show that potential users of a communication BCI reported to prefer an active rather than reactive strategy for BCI control.

Moreover, it should be considered that there is a high variety between (prospective) users both in terms of relevant neurological characteristics (e.g., because of underlying condition or disease progression) as well as personal needs and wishes. Therefore, there will be no one-size-fits all solution in speech-BCI design, but it also means that changes may occur for individual users. Sankaran et al. (2023) therefore argue for the customization of speech-BCIs to meet preferences and needs of individual users on various aspects, including the relative weight given to the language model in decoding. Klein et al. (2022) also argue that in the development of personalized language models, for instance, feedback of users on ethical concerns regarding storage and use of these models should be considered. Liberati et al. (2015) further showed that potential users reported the need for a system that could be adapted to the individual as their disease progresses, so that the system could support them throughout different stages of ALS. Additionally, several calls have been made to allow for customization of the voice that is used in the BCI system (Nathanson, 2017; Luo et al., 2022; Sankaran et al., 2023). To address this need, voice bank strategies have been proposed for the creation of a personalized synthetic voice that approximates a person’s natural voice (Yamagishi et al., 2012; Veaux et al., 2013; Cave and Bloch, 2021). A recent study by Lu et al. (2023) has however shown that decoding of neural signals for tone may also become possible, further increasing options for tonal control.

In considering these trade-offs, it may also be imagined that the aim of speech-BCIs varies between contexts, also influencing the relative importance of (semantic) accuracy and user-control. Speech-BCI could also accommodate such dual use by incorporation of context-specific settings. For example, in everyday practical interactions (e.g., when getting ready for the day with the help of a care partner), functional and fast (and thus less controlled) speech may be sufficient, or even preferred. Contrastingly, in a setting in which a user aims to discuss their considerations on providing informed consent for a medical procedure, a much higher-level of control would be preferred, even if this is at the cost of some speed. Allowing users to switch between these settings would enhance their overall control and autonomy.

Additionally, it is also important to recognize that in addition to design solutions, there may also be non-technical ways to address some of these ethical considerations and improve user control. To start, users may also improve their control over externalized speech by becoming more skilled in their use. Skills for guidance control over speech-BCIs could for instance also mean that the user learns to work with any limitations of the device, for instance by making deviations from their intended speech by emphasizing sounds or adjusting their choice of words to reach a desired output (Rainey, 2023).

Moreover, to address some of the ethical implications of reduced user-control in speech-BCIs, conversation partners may also contribute to the correct interpretation of users’ speech intentions through conversation strategies (e.g., asking additional questions to validate certain answers) (Birbaumer et al., 2014). This way conversation partners share in the interpretive burden and the *working with the device*. Conversation partners may also contribute to addressing issues for user’s self-representation by adjusting their expectations of BCI-mediated speech by considering the level of control users have over the output (Rainey et al., 2019). By recognizing possible limitations of speech-BCIs and not unequivocally taking BCI-mediated speech as that of the user, conversation partners could leave room for future corrections by users. At the same time however, such adjusted expectations should not lead conversation partners to question or refuse to accept user’s externalized speech, even after repeated confirmation.

### 5.2 Need for conceptual clarity

Our review showed that in discussions on ethics of user-control in speech-BCIs a variety of terminology is used to describe essential concepts in this discussion.

To start, we showed that user-control is described in several ways throughout literature, broadly divided in executory and guidance control. Yet we also showed that how such control mechanisms (especially for guidance control) could be embedded in the BCI-mediated speech production process remains underdetermined. Further specification of these mechanisms and their place in the BCI-mediated speech production process could provide a useful framework for continued ethical analysis. Yet, this would first require a comprehensive model of the BCI-mediated speech production process itself, which may have relevant differences from natural speech production and may vary between different types of speech-BCIs. To our knowledge, no such model has yet been developed.

Additionally, the variety of terminology also included several ethical concepts related to different aspects of autonomy. The included literature showed ambiguity as to how the specific ethical concepts can be defined, how they interrelate, are distinct from one-another, or where they are or can be used interchangeably. Equally addressing this variety of ethical concepts discussed in this neuroethics debate, Schönau et al. (2021) argue that, rather than considering these ethical dimensions (including responsibility, privacy, authenticity or trust) separately, their mutual significance could be best captured “under a unified heading of agency” (p. 1). In order to gain clarity on how ethical concepts in discussions on speech-BCIs can be conceptualized in relation to agency, Schönau et al. (2021) provide a comprehensive overview.

In a similar way, we have observed that in literature on speech-BCIs terminology aimed to reflect ‘inner speech’ varies across literature, both for inner speech that is intended and not intended for externalization. Terminology for inner speech intended for externalization included covert speech (Rainey et al., 2020a; Maslen and Rainey, 2021; Rainey, 2023), attempted speech (Moses et al., 2021; Branco et al., 2023; Sankaran et al., 2023) and imagined speech (Rainey et al., 2020a; Rainey, 2023; Sankaran et al., 2023), while inner speech not intended for externalization was described to include, inner monologue (Maslen and Rainey, 2021), internal speech (Sankaran et al., 2023), and private thought, practice speech and mind-wandering (Rainey et al., 2019). Alderson-Day and Fernyhough (2015) who define inner speech as “the subjective experience of language in the absence of overt and audible articulation” (p. 934), also report this high diversity in terminology to reflect variations of inner speech in broader academic literature (i.e., not limited to speech-BCIs). We argue however that in the context of ethics and design of speech-BCIs, clear terminology is needed to be specific on what type of inner speech is discussed, as externalization of these different types of inner speech can have different ethical implications. We here argue that ‘attempted speech’ is best suited to describe motor-intentions for speech, as it clearly describes the intention for production (i.e., externalization) of speech, rather than the more ambiguous terms of inner, imagined, or covert speech, that lack this clear description of intention for production. We argue it to be important to maintain these distinctions in terminology to not inflate any ethical concerns that may exist for other types of inner speech but are not relevant for attempted speech. To note however is that while current state-of-the-art speech-BCIs utilize such motor intentions for speech, this may not always be the case in the future. New types of speech-BCIs using other neural signals may require reconsideration of such terms.

### 5.3 Rate of technological development and ethical inquiry

It is important to recognize that the field of BCI research is evolving fast. Being so inextricably linked with technological and design choices, ethical implications of speech-BCIs may be expected to change alongside these technological advances. Therefore, when reflecting on ethical aspects of speech-BCIs it remains important to take into consideration the latest technological advances, to prevent inflation or undue continuation of ethics concerns that have been addressed through design or ignore new ethical implications that have arisen. One way of doing so is to use the ethics parallel research approach as suggested by Jongsma and Bredenoord (2020), in which ethicists work as embedded ethicists in close collaboration with researchers and developers.

## 6 Strengths and limitations

As ethics literature specifically addressing user-control in speech-BCIs is still limited, a small number of seminal articles and scholars dominate these discussions. Still, these seminal articles provide useful suggestions of how user-control in speech-BCIs could be conceptualized from an ethics perspective. Using a narrative approach we have aimed to relate these concepts to broader literature, also linking this to more practical notions and responses. As this field has developed over the past decades, we consider this paper to provide a valuable overview of current discussions on ethical aspects of user-control in speech-BCIs, as well as a starting point for further inquiry in the ethical significance as well as practical feasibility of embedding mechanisms for user-control in (continuous) speech-BCIs.

## 7 Conclusion

We conclude that though user-control in speech-BCIs may address ethical implications of this technology, including relevant aspects of user’s autonomy and responsibility for BCI-mediated speech, additional research is required to find out how mechanisms for user-control may be translated to speech-BCI design. Additionally, we argue that considerations on mechanisms for user-control in speech-BCI require us to address whether the aim of speech-BCIs should be considered expressive or instrumental as this likely has implications for the relative importance of user-control in these devices. These aims may also be situational and personal to specific users. We also flag the need for conceptual clarity in the ethical discourse on (user-control in) speech-BCIs, as different forms of inner speech can have distinct ethical implications, and different, though related, ethical concepts can have specific implications for speech-BCI design.

## Author contributions

OvS: Conceptualization, Data curation, Investigation, Methodology, Writing – original draft, Writing – review & editing. DS: Data curation, Investigation, Writing – original draft, Writing – review & editing. MV: Writing – original draft, Writing – review & editing. MB: Supervision, Writing – original draft, Writing – review & editing. KJ: Conceptualization, Investigation, Supervision, Writing – original draft, Writing – review & editing.

## Footnotes

^1^We focus our current analysis on spoken speech-BCIs, yet some ethical considerations may also be relevant for written speech-BCIs.
